# Evidence for one-dimensional chiral edge states in a magnetic Weyl semimetal Co_3_Sn_2_S_2_

**DOI:** 10.1038/s41467-021-24561-3

**Published:** 2021-07-13

**Authors:** Sean Howard, Lin Jiao, Zhenyu Wang, Noam Morali, Rajib Batabyal, Pranab Kumar-Nag, Nurit Avraham, Haim Beidenkopf, Praveen Vir, Enke Liu, Chandra Shekhar, Claudia Felser, Taylor Hughes, Vidya Madhavan

**Affiliations:** 1 Department of Physics and Materials Research Laboratory, University of Illinois Urbana-Champaign, Urbana, IL USA; 2 Condensed Matter Physics Department, Weizmann Institute of Science, Rehovot, Israel; 3 Max-Planck-Institute for Chemical Physics of Solids, Dresden, Germany; 4 Department of Physics and Institute for Condensed Matter Theory, University of Illinois at, Urbana-Champaign, Urbana, IL USA

**Keywords:** Electronic properties and materials, Topological insulators

## Abstract

The physical realization of Chern insulators is of fundamental and practical interest, as they are predicted to host the quantum anomalous Hall (QAH) effect and topologically protected chiral edge states which can carry dissipationless current. Current realizations of the QAH state often require complex heterostructures and sub-Kelvin temperatures, making the discovery of intrinsic, high temperature QAH systems of significant interest. In this work we show that time-reversal symmetry breaking Weyl semimetals, being essentially stacks of Chern insulators with inter-layer coupling, may provide a new platform for the higher temperature realization of robust chiral edge states. We present combined scanning tunneling spectroscopy and theoretical investigations of the magnetic Weyl semimetal, Co_3_Sn_2_S_2_. Using modeling and numerical simulations we find that depending on the strength of the interlayer coupling, chiral edge states can be localized on partially exposed kagome planes on the surfaces of a Weyl semimetal. Correspondingly, our d*I*/d*V* maps on the kagome Co_3_Sn terraces show topological states confined to the edges which display linear dispersion. This work provides a new paradigm for realizing chiral edge modes and provides a pathway for the realization of higher temperature QAH effect in magnetic Weyl systems in the two-dimensional limit.

## Abstract (web summary)

Magnetic Weyl semimetals in the 2D limit may behave like 2D Chern insulators and host the quantum anomalous Hall effect at high temperatures. Here, the authors report the observation of linearly dispersing topological states confined to the edges of the kagome Co_3_Sn terraces in the magnetic Weyl system Co_3_Sn_2_S_2_.

## Introduction

**Fig. 1:**
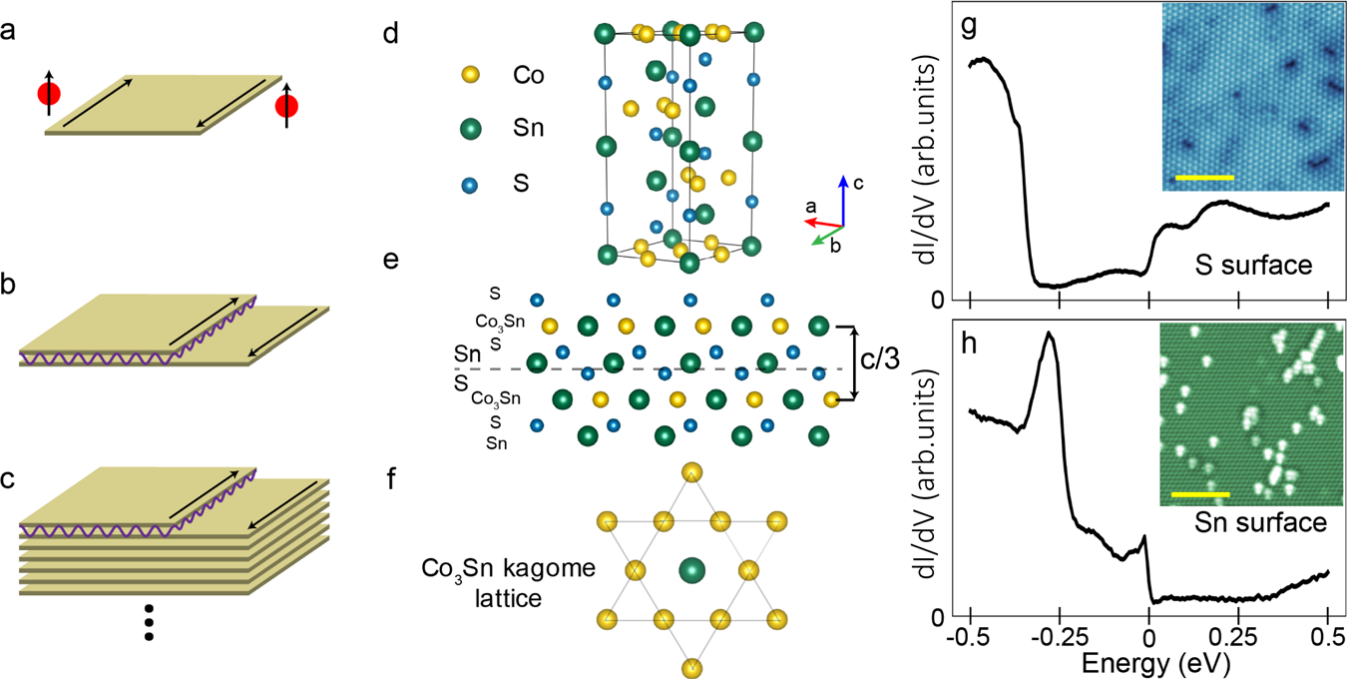
Crystal structure, topography, and spectroscopy of Co_3_Sn_2_S_2_ . **a** Single Chern insulator, with one chiral edge state. **b** Chern insulator bilayer system with step geometry and inter-layer coupling (represented by the purple lines).When the coupling is strong enough to turn the bilayer system trivial, chiral edge states appear at the edges of the exposed planes as well as at the interface of the bilayer and single layer. **c** Large stack of Chern insulators with intermediate inter-layer coupling to form a Weyl semimetal. For large regions of parameter space, the Weyl semimetal phase coincides with the conditions necessary for the counter-propagating chiral edge states seen in **b** (see Supplementary Note 1). **d** Unit cell of Co_3_Sn_2_S_2_. Sn is represented by a large green sphere, Co by a medium yellow sphere, and S by a small blue sphere. **e** Several layers of the crystal as viewed from the (100) plane. Cleavage occurs between the Sn and S layers, as indicated by a dashed line. **f** Schematic of the kagome structure present in the Co_3_Sn plane with Co forming 6 triangles surrounding a central Sn atom. **g** Spectra typical of S surface (*I* = 300 pA, *V* = 500 mV). Inset is 30 nm × 30 nm topography showing vacancies typical of S surface (*I* = 30 pA, *V* = 500 mV). The scale bar in the inset is 10 nm. **h** Spectra typical of Sn surface (*I* = 60 pA, *V* = 500 mV). Inset is 30 nm × 30 nm topography showing adatoms typical of Sn surface. (*I* = 110 pA, *V* = 80 mV). The scale bar in the inset is 10 nm.

The quantized Hall conductance of the quantum Hall effect is a striking example of the macroscopic consequences of quantum phenomena^1^. In the quantum Hall effect, large magnetic fields generate Landau levels in a two-dimensional (2D) material. The Landau levels acquire a non-zero topological index, resulting in chiral edge currents that are a manifestation of the quantized Hall response. Haldane’s conception of the Chern insulator^2^, or quantum anomalous Hall insulator^3^, takes this idea a step further. A Chern insulator is a 2D material that exhibits the quantum Hall effect in the absence of an external magnetic field. The distinctive features of Chern insulators are their quantized Hall conductance, and topologically protected chiral edge states^4^, which travel in unidirectional channels (see Fig. 1a). The transport signatures of the quantum anomalous Hall effect (QAHE) were originally reported in a 2D magnetically doped topological thin film^5^, and advancements in QAHE signatures in magnetically doped topological insulators^6–13^ have recently been extended to intrinsic magnetic topological insulators^14^ and twisted bilayer graphene^15^. While these results are breakthroughs in studying QAHE, future progress in studying chiral edge states is limited by the low Curie temperatures (<30 K) of these material systems and the complex heterostructures often necessary to realize chiral edge states.

Interestingly, Chern insulators are also related to a variety of higher-dimensional topological systems, not least of which are the magnetic Weyl semimetals (WSMs). One can in fact model magnetic WSMs as layers of 2D Chern insulators that are coupled in the stacking direction^16,17^. Thus, an unexplored route to 2D Chern insulators is to identify a three-dimensional (3D) layered magnetic Weyl semimetal fitting this description. Recent developments in candidate magnetic WSMs^18,19^ now provide a promising alternative arena for the study of chiral edge states. In the spirit of the coupled-layer model presented by Balents and Burkov^16^, we can show that stepped terraces on the surface of a WSM can harbor chiral edge states localized on the steps. First, we note that Weyl semimetals are an intermediate critical phase between a trivial insulator and a magnetic weak topological insulator, the latter of which is adiabatically connected to a decoupled stack of Chern insulators^20–22^. Importantly, the two gapped phases and the intermediate gapless phase can be reached by starting with decoupled layers of Chern insulators and increasing the strength of the inter-layer coupling. To illustrate, take a bilayer of Chern insulators both having the same non-zero Chern number. If the coupling between the two layers is increased, the system will eventually undergo a transition where the strong tunneling creates a trivial phase of the bilayer with a vanishing Chern number. Interestingly, if we strip off a part of one of the layers as depicted in the schematic in Fig. 1b, the exposed single-layer region will revert to a non-trivial Chern insulator since it was the inter-layer coupling that drove it to be trivial. Thus, both the end of the single layer and the single-step defect itself will harbor a chiral edge state (see Supplementary Note 1 for more details). The existence of the edge state on the end of the single layer is obvious, but the edge state on the step defect appears only because the remaining bilayer region with strong tunneling is a trivial insulator. Thus, this process serves to expose a region of the Chern insulator despite the full bilayer system being trivial. The concept of exposing topological sub-systems when the combined system is trivial was explored in two recent papers^23,24^ in the context of a bulk topological proximity effect, and embedded topological insulators, respectively.

While a single bilayer of Chern insulators is not sufficient to realize the intermediate WSM phase between the topological and trivial regimes, we extend this analysis to the case of many layers (as shown schematically in Fig. 1c) to model the properties of a WSM (Supplementary Note 1). We find that step-localized chiral edge states continue to exist in WSMs in a wide swath of the topological phase diagram that is parameterized by the Chern-insulator gap of decoupled QAH planes and inter-layer tunneling (see Supplementary Note 1). Indeed, we find that whenever the surface terrace exhibits the localized chiral modes on the steps, the system is in a WSM phase. Thus, while not every Weyl semimetal will harbor step-localized chiral modes, a large fraction do. One can therefore be optimistic that, given a magnetic WSM, there is a large probability that terraces will exhibit localized QAH regions and manifest localized chiral modes.

To investigate these theoretical predictions, we study the magnetic Weyl semimetal Co_3_Sn_2_S_2_. There is substantial prior evidence for the topological WSM nature of this material, including a large anomalous Hall effect^25,26^, signatures of Fermi arc states in STM^27–29^, and flat band diamagnetism caused by Berry curvature^30^. Importantly, the compound is predicted to host the QAHE in the 2D limit^31^, and models of a single, magnetic Co_3_Sn kagome layer predict a non-zero Chern number^30^. Therefore, this material appears to fit the model of a WSM constructed from stacked and coupled Chern insulators. In addition, the material’s high Curie temperature of 170 K^32^ makes Co_3_Sn_2_S_2_ an ideal candidate to test the predictions of terraces within the coupled Chern insulator model. If edge states are observed, then it would provide strong experimental evidence that the 2D limit of this material may host the QAHE at elevated temperatures.

## Results

### Topography and spectroscopy of S-surfaces and Sn-surfaces

We use scanning tunneling microscopy and spectroscopy (STM/S) at 4 K to investigate the possibility of realizing chiral edge states in Co_3_Sn_2_S_2_. Co_3_Sn_2_S_2_ is a layered material consisting of a kagome Co_3_Sn plane in-between two hexagonal S layers, and all sandwiched in between two hexagonal Sn layers (Figs. 1d to 1f). The hexagonal lattice constant is *a* = 5.3 Å, while three stackings of Sn-S-Co_3_Sn-S-Sn layers each translated by (1/3, 1/3, 1/3) construct a full unit cell, giving a lattice constant *c* = 13.2 Å^19^. The material is a half-metallic ferromagnet, with magnetic properties derived from the moments of the Co atoms aligned along the c-axis^18,32^.

Co_3_Sn_2_S_2_ bulk single crystals cleaved along the (001) direction most often expose two distinct surfaces^28–30^ that are both hexagonal in nature. This suggests that the main cleavage plane is between the S-Sn/Sn-S layers revealing either the Sn or S layer. A third possible termination is a honeycomb-like kagome Co_3_Sn plane which is rare. The two surfaces most seen in STM can be distinguished topographically (one consisting of vacancies and the other adatoms) and also spectroscopically (as shown in Figs. 1g and 1h). On the surface with adatoms, we observe a sharp peak in the spectra near −12 meV associated with a diamagnetic flat band^30^, along with another large peak near −300 meV. On the surface with vacancies, we see a depression in the density of states from −300 meV to 0 meV, with two broad peaks occurring at 50 meV and 200 meV, respectively.

Previous STM studies have arrived at different conclusions on the chemical identification of these two common hexagonal surfaces^28–30^. To reconcile this issue, we utilize the symmetry of the local density of states signatures of defects to identify the termination layer. This method has been used successfully for the chemical identification of surface lattices of other layered materials^33^. The density of state signatures of defects centered at positions that do not correspond to the positions of the top layer atoms can be attributed to defects in the layer below (DLB). Such signatures are present in both commonly observed hexagonal surfaces. However, DLBs existing on the surface with vacancies have a reduced symmetry (a triangle with one bright vertex) than the symmetry of DLBs on the surface with adatoms (a clover with equally bright vertices). The reduced symmetry of DLBs seen on the surface with vacancies is consistent with the layer below being the Co_3_Sn kagome plane, which has the same reduced symmetry compared to hexagonal layers. This allows for the identification of the surface with vacancies as being the S plane, and the surface with the adatoms being the Sn plane (see Supplementary Note 2) which is consistent with recent STM studies^34^.

### Edge modes on Co_3_Sn step edges

**Fig. 2:**
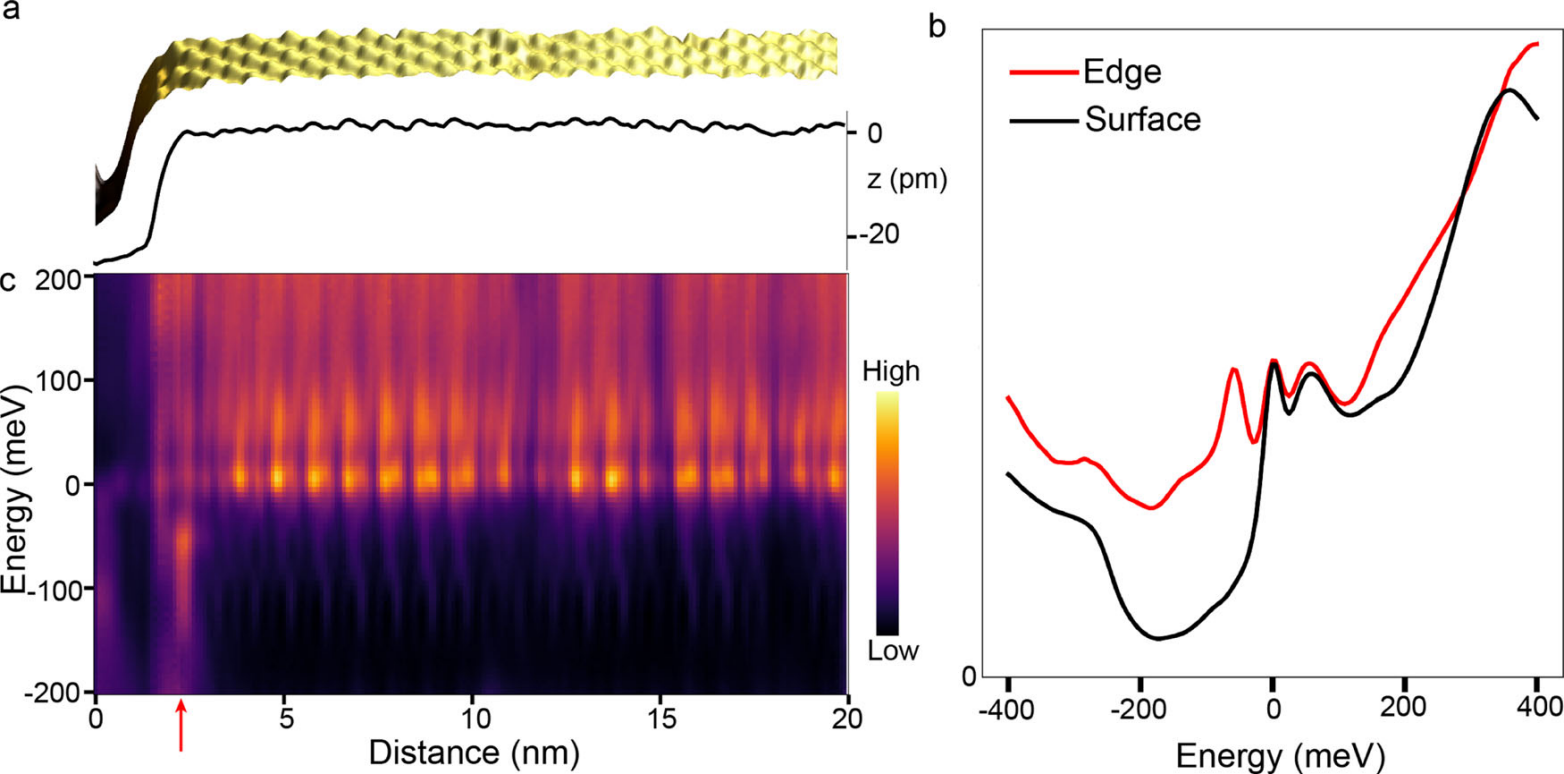
Spectroscopic data showing edge state on Co_3_Sn plane. **a** Three-dimensional topography and line profile of an exposed Co_3_Sn surface. Note that only part of the step is shown here. The atomic-scale features represent a cluster of three nearest neighbor Co-atoms. The full step and zoomed-out image are shown in Supplement Fig. 4. **b** Spectroscopic heatmap of the differential conductance along the line profile shown in **a**. An edge state is clearly seen as indicated by the red arrow. The state is confined to the edge with an extent of approximately 1.5 nm transverse to the step edge. **c** Spectra on the Co_3_Sn plane taken at the edge (red) and away from the edge on the surface (black). A noticeable peak in the density of states at the edge exists at −60 meV along with a broad increase in the density of states below the Fermi energy, indicative of the edge mode. The color indicates the value of DOS. Spectra and topography were obtained with setpoint current *I* = 150 pA and voltage *V* = 70 meV.

To look for edge modes, we investigate large Co_3_Sn terraces terminated by a step edge (Fig. 2a). The Co_3_Sn surface can be distinguished spectroscopically from the S and Sn planes. From Fig. 2b, the spectra seen on the Co_3_Sn surface have two sharp peaks in the density of states at 0 meV and +60 meV which are not seen in the spectra on either the S and Sn surfaces and are consistent with spectra previously reported on the Co_3_Sn surface^28,29^. Point spectra taken on the edge of this surface reveals a broad accumulation density of states that is not present in spectra taken away from the edge. The broad accumulation of density of states at the edge over these energies is unique to the Co_3_Sn surface (see Supplementary Fig. 3). Taking spectra along the line profile shown in Fig. 2a reveals that this enhanced density of states is localized to the edge, with an extent of approximately 1.5 nm transverse to the step edge (Fig. 2c and Supplementary Fig. 4). The highly localized nature of these states suggests that they can be attributed to an edge mode existing on an exposed Co_3_Sn plane. Interestingly, despite the presence of impurities on the edge which act as scattering centers, we find no sign of quantization in the d*I*/d*V* spectra of the edge mode (see Supplementary Fig. 4). This however changes markedly when we investigate narrow terraces, as we show next.

### Linearly dispersing edge states on narrow Co3Sn terraces

**Fig. 3:**
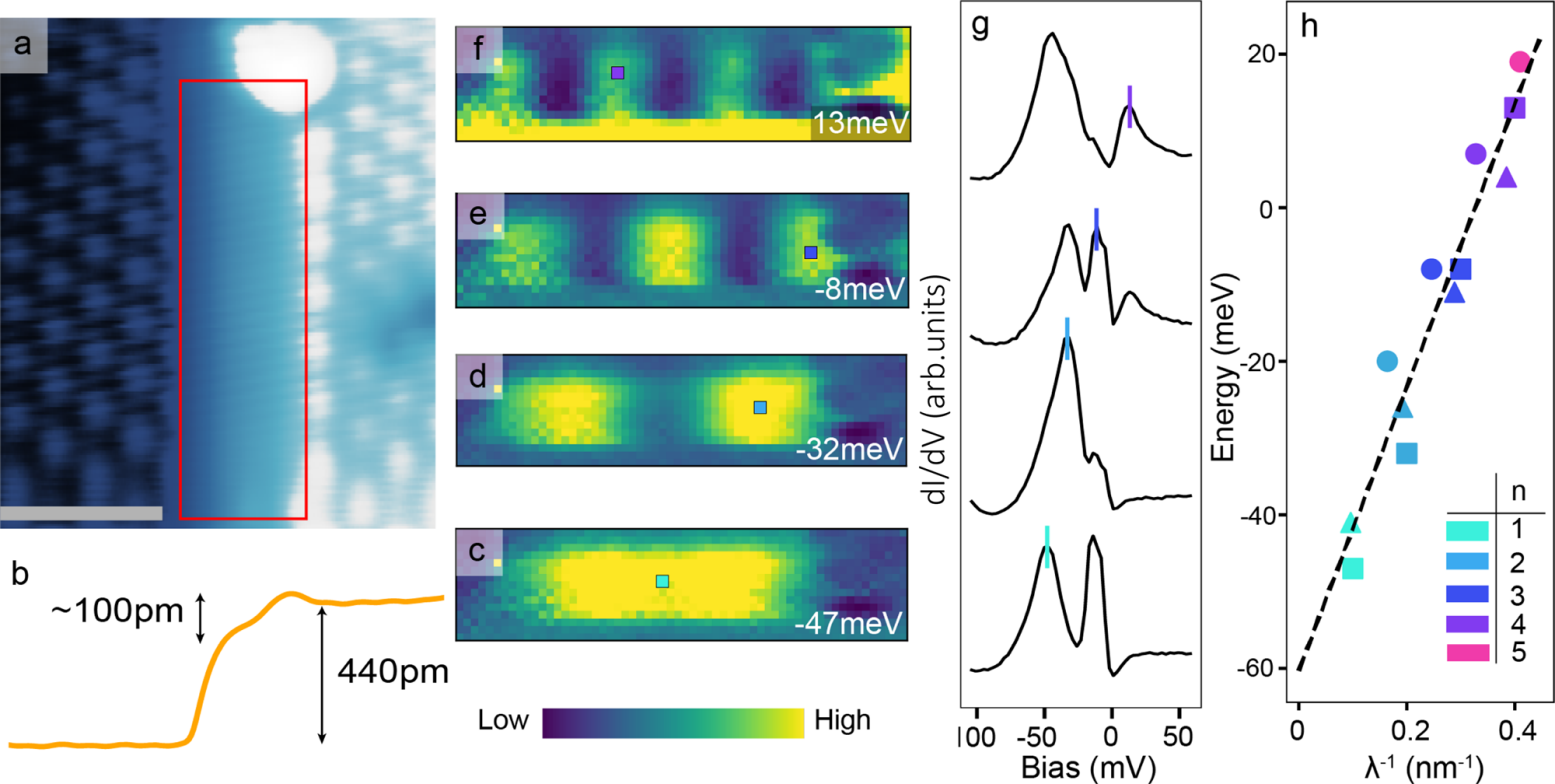
Observation of linearly dispersing bound states on Co_3_Sn Kagome terrace. **a** Topography of a step edge between two S surfaces containing a small terrace of the Co_3_Sn plane, indicated by the red rectangle. (*I* = 20 pA, *V* = −200 mV). The gray inset scale bar indicates 2 nm. **b** Line profile of the step edge. Total step height is consistent with one-third of a lattice constant *c* or approximately 440 pm. The terrace is approximately 130 pm below the top S surface. **c**–**f** Density of state maps of the region enclosed by the red rectangle in **a** at energies for each quantum well like state. The color indicates the value of DOS. (*I* = 200 pA, *V* = −200 mV). **g** Spectra at locations of the high density of states for each quantum well-like state indicated by small squares in **c**–**f** starting at *n* = 1 at the bottom to *n* = 4 at the top. Spectra are offset for clarity. A small vertical line indicates the peak associated with the energy of the state. **h** Energy versus inverse wavelength relation for states found on three Co_3_Sn terraces. The states shown in the figure are represented by squares, while states from the two other terraces are circles and triangles. The linear dashed line is provided as a guide to the eye. The inverse wavelength is calculated assuming the distance between peaks is one wavelength.

To further explore the nature of these edge states, we study narrow terraces of the kagome Co_3_Sn planes. Incomplete cleavage between Sn and S layers occasionally exposes small terraces near step edges on the S surface (Fig. 3a and Supplementary Fig. 5 and 6). These terraces are approximately 130 pm below the S surface (Fig. 3b). As shown in the extended data (Supplementary Fig. 7), the closest plane to S is the Co_3_Sn plane at a height of 156 pm below the Sn surface with the next plane being 312 pm below. The thin terrace can thus be unambiguously identified as the Co_3_Sn plane. Taking a d*I*/d*V* map of one of these terraces reveals striking features, highlighted by quantum well-like bound states at various energies shown in the DOS images in Fig. 3c, f. Note that these states are responsible for obscuring the atomic resolution on this terrace. The number of nodes increases with energy, indicating a positive dispersion. The bound state energies for each distinct quantum well-like state can be identified from peaks in the spectra at locations where the density of states is maximum (see Fig. 3g). In the terrace of length 5.2 nm, shown in Fig. 3a, we observe a sequence of four quantum well like states ranging from *n* = 1 to *n* = 4, while in the other two terraces, with lengths of 5 nm and 6.1 nm as shown in Supplementary Fig. 3, we observe a sequence of four and five quantum well like states, respectively. The notation for the *n*th state is the same as in the classic quantum well, where *n* indicates a bound state with *n* + 1 nodes and *n* maxima in the local density of states. Converting the linear energy dependence on the bound state wavelength into a dispersion velocity we find a value of approximately 5 × 10^4^ m/s. Quantum well like one dimensional (1D) states have been observed previously with STM in systems such as Au/Cu adatom chains and semiconductor terraces^35–39^, however all of these studies show a quadratic dependence of the bound state/sub-band energy on the number of nodes, as expected from the conventional quantum well states originating from free-electron-like quadratic dispersion. Unlike these examples, the dispersion seen on all the terraces we observe is linear (as plotted in Fig. 3h).

### Numerical simulation of hybridized chiral edge states

**Fig. 4:**
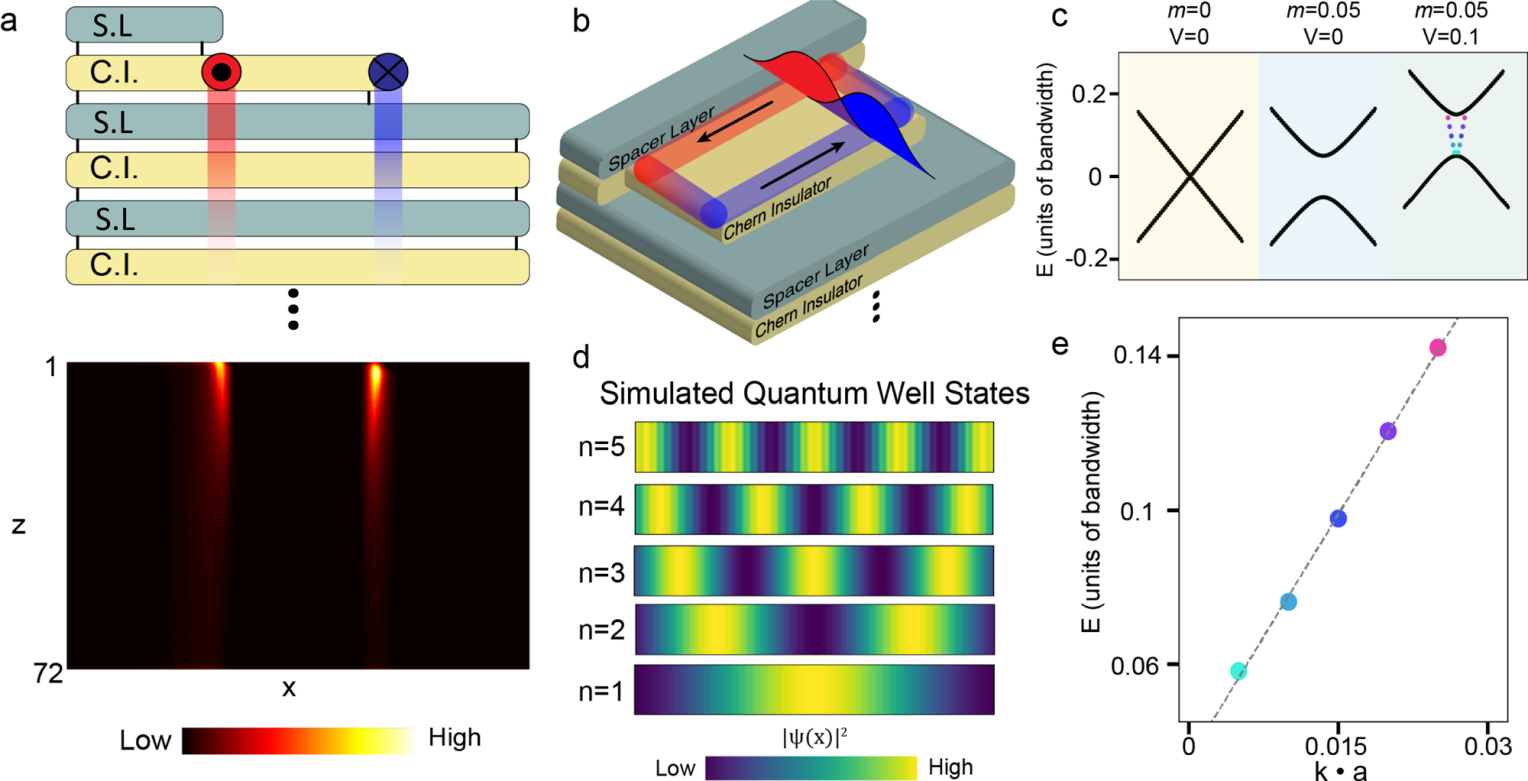
Tight binding calculation of linearly dispersing states within a potential well. **a** Schematic of coupled Chern insulator layers (C.I.) with alternating spacer layers (S.L) , with a Chern insulator terrace (top panel), a simple stack that mimics our geometry. The red and blue circles indicate 1D chiral modes traveling out of and into the page, respectively, with the red and blue trails indicating their power-law decay into the bulk. The bottom panel is a numerical simulation of 70 full Chern insulator layers plus two partial layers as shown in the top panel. The bright regions in the bottom panel show the numerically calculated chiral edge states on the exposed terrace and their decay into the bulk. **b** Schematic of the narrow, confined Co_3_Sn terraces. The exposed Chern insulator layer (Co_3_Sn plane) hosts chiral edge states, and when the terrace is sufficiently narrow, the two counterpropagating states will overlap and interact, as shown by the red and blue curves. **c** Low energy dispersion for different parameters of *m* and *V*. With no mixing or potential well, two linearly dispersing bands cross at zero momentum (light yellow left panel). Adding a mixing term of five percent of the bandwidth, a gap opens as the two bands hybridize (light blue middle panel). Further adding the potential of ten percent of the bandwidth increases the energy of all states and causes five states to be confined entirely within the potential well (light green right panel), indicated by the colored dots within the gap. **d** Wavefunctions of the confined, linearly dispersing states shown in **e**. **e** Dispersion of the states confined within the potential well from the light green panel in **c**. A straight dashed line is provided as a guide to the eye.

To interpret the STM results using the layered WSM model (Supplementary Note 1) we first perform numerical simulations of a WSM having a surface terrace with a partially exposed Chern insulator plane as shown in Fig. 4a top panel. Spacer layers between Chern insulator layers, in this case, the S and Sn layers, effectively change the coupling in the stacking direction. Identifying S/Sn as spacer layers is consistent with earlier work that shows that the band structure near the Fermi energy mainly arises from the Co-3d bands^26^, as well as work showing that the Kagome monolayer alone is a Chern insulator^30^. Remarkably, we find that the chiral modes that we argued are present in the strongly coupled bilayer are recovered in the WSM regime, as seen in Fig. 4a bottom panel. These modes counter-propagate and are localized at edges of the partially exposed Chern insulator planes and decay exponentially along the surface and as a power-law into the bulk.

After confirming the existence of the chiral terrace modes within the coupled Chern insulator model, we further simulate the DOS signatures of the two (counter-propagating) chiral edge states in a quantum well using a simple one-dimensional lattice model for linearly dispersing, counter-propagating modes in a potential well. We use a one-dimensional Hamiltonian of the form $H\,=\,\mathop{\sum}\nolimits_{n}\big(\frac{i}{2}{c}_{n+1,{{\alpha }}}^{\dagger }{c}_{n,{{\beta }}}{{{\sigma }}}_{{\rm{\alpha }}{\rm{\beta }}}^{z}\,-\,\frac{i}{2}{c}_{n,{{\alpha }}}^{\dagger }{c}_{n+1,{\rm{\beta }}}{{\rm{\sigma }}}_{{\rm{\alpha }}{{\beta }}}^{z}\,+\,m{c}_{n,{{\alpha }}}^{\dagger }{c}_{n,{{\beta }}}{{{\sigma }}}_{{{\alpha }}{{\beta }}}^{x}+V\left(n\right){c}_{n,{{\alpha }}}^{\dagger }{c}_{n,{{\beta }}}{{{\delta }}}_{{{\alpha }}{{\beta }}}\big)$. Here the operator ${c}_{n,{\rm{\alpha }}}^{\dagger }$ creates an electron on site *n* in orbital α, the parameter *m* represents the evanescent hybridization between the counter-propagating modes,$V$(*n*)is a discretized finite square well potential, and all energy scales are in units of the tunneling strength which we have set to unity. See Fig. 4a, b and Supplementary Fig. 8 for a schematic illustration of the model setup. In the absence of the square well potential, this Hamiltonian yields an energy spectrum $E=\pm \sqrt{{{\sin }}{\left({{\bf{k}}}_{a}\right)}^{2}+{m}^{2}}$ which, for vanishing hybridization (*m* = 0) and long wavelengths (**k**_*a*_«1), recovers the linear dispersion $E=\pm \left|{\bf{k}}\right|$ (see the leftmost panel in Fig. 4c and Supplementary Fig. 8). Turning on the hybridization term *m* opens a gap, lifting the degeneracy at zero momentum as shown in the middle panel of Fig. 4c. The length of the terrace is modeled as a finite square well, *V*(*n*), with a width that is a small fraction of the total system size. Adding a finite potential well with a non-zero mixing term creates eigenstates within the hybridization-induced energy gap, as illustrated in the rightmost panel in Fig. 4c. The electron density of the eigenstates confined to the potential well resembles the bound states observed in our experiment (see Fig. 4d). In addition, for a wide range of *m*, the energies of the confined states are linearly dependent on the confinement quantum number *n* (see Fig. 4e and Supplementary Fig. 8). Also, if *m* becomes too large, the dispersion relation begins to resemble a typical quadratic band and the bound state energies cross over to a quadratic dependence on the confinement quantum number. If *m* is too small the counter-propagating modes will not effectively form a bound state, e.g., if the plateau is very wide so that the evanescent coupling between the opposed edge modes is small, the chiral edge mode will hit the potential wall and turn to continue its circulation around the plateau boundary instead of forming a coherent bound state. Physically, this indicates that when the terrace width is small enough to hybridize chiral edge states, we should expect quantum well-like states to develop. Notably, the transverse extent of the edge state observed on the Co_3_Sn surface is of the same order (~1.5 nm) as the width of terraces containing quantum well-like bound states (Fig. 2 and Supplementary Fig. 5). This suggests that the edge states are within the intermediate evanescent mixing regime necessary to observe linearly dispersing bound states.

## Discussion

We have found that our model can reproduce all the features seen in our experiment, providing a clear, self-consistent explanation for the existence of linearly dispersing quantum well-like bound states, composed of hybridized chiral edge states on an exposed kagome Co_3_Sn terrace. While the chiral nature of these states cannot be directly probed with STM, it follows naturally from topological edge states within a material with broken time-reversal symmetry. It is worth noting that similar interference patterns of topological edge states have been observed by STM in Bi single crystals^40,41^. While Bi edge state interference patterns are due to intra-band scattering of topological edge states, our result originates from chiral edge states with the same quantum numbers that couple because of their close spatial proximity.

This observation of chiral edge modes within a bulk magnetic Weyl semimetal provides evidence for the physical realization of the model presented by Balents and Burkov^16^. The modification of this original model to terrace geometries, combined with our experimental observations, theorizes a new paradigm for studying chiral edge states in a wide range of magnetic Weyl semimetals via local probes without requiring thin film growth or heterostructures. Most importantly, a reductionist approach to this model suggests that a material fitting this description will, in the 2D limit, be a Chern insulator hosting the QAHE. Co_3_Sn_2_S_2_’s high Curie temperature^32^ which persists into the 2D limit^42^, existing theoretical calculations in the 2D limit^31^, and our observation of linearly dispersing chiral edge modes make the 2D limit of Co_3_Sn_2_S_2_ a strong candidate for the observation of intrinsic QAHE at elevated temperatures.

## Supplementary information

Supplementary Information

## Data Availability

The data in this work will be made available at reasonable request. Correspondence and requests for materials should be addressed to V.M. or H.B.

## References

[1] Klitzing KV, Dorda G, Pepper M (1980). New method for high-accuracy determination of the fine-structure constant based on quantized hall resistance. Phys. Rev. Lett..

[2] Haldane FDM (1988). Model for a quantum hall effect without Landau levels: condensed-matter realization of the ‘parity anomaly’. Phys. Rev. Lett..

[3] Laughlin RB (1983). Anomalous Quantum Hall effect: an incompressible quantum fluid with fractionally charged excitations. Phys. Rev. Lett..

[4] Halperin BI (1982). Quantized Hall conductance, current-carrying edge states, and the existence of extended states in a two-dimensional disordered potential. Phys. Rev. B.

[5] Chang C-Z (2013). Experimental observation of the Quantum anomalous Hall effect in a magnetic topological insulator. Science.

[6] Götz M (2018). Precision measurement of the quantized anomalous Hall resistance at zero magnetic field. Appl. Phys. Lett..

[7] Rosen IT (2017). Chiral transport along magnetic domain walls in the quantum anomalous Hall effect. npj Quantum Mater..

[8] Jotzu G (2014). Experimental realization of the topological Haldane model with ultracold fermions. Nature.

[9] Bestwick AJ (2015). Precise quantization of the anomalous Hall effect near zero magnetic field. Phys. Rev. Lett..

[10] Chang C-Z (2015). High-precision realization of robust quantum anomalous Hall state in a hard ferromagnetic topological insulator. Nat. Mater..

[11] Kou X (2014). Scale-invariant quantum anomalous Hall effect in magnetic topological insulators beyond the two-dimensional limit. Phys. Rev. Lett..

[12] Liu M (2016). Large discrete jumps observed in the transition between Chern states in a ferromagnetic topological insulator. Sci. Adv..

[13] Allen M (2019). Visualization of an axion insulating state at the transition between 2 chiral quantum anomalous Hall states. Proc. Natl Acad. Sci. USA.

[14] Deng, Y. et al. Quantum anomalous Hall effect in intrinsic magnetic topological insulator MnBi2 Te4. *Science* eaax8156 10.1126/science.aax8156. (2020). 10.1126/science.aax8156 31974160

[15] Serlin, M. et al. Intrinsic quantized anomalous Hall effect in a moiré heterostructure. *Science* eaay5533 10.1126/science.aay5533 (2019). 10.1126/science.aay5533 31857492

[16] Burkov AA, Balents L (2011). Weyl semimetal in a topological insulator multilayer. Phys. Rev. Lett..

[17] Armitage NP, Mele EJ, Vishwanath A (2018). Weyl and Dirac semimetals in three-dimensional solids. Rev. Mod. Phys..

[18] Dedkov YS, Holder M, Molodtsov SL, Rosner H (2008). Electronic structure of shandite Co3Sn2S2. J. Phys..

[19] Vaqueiro P, Sobany GG (2009). A powder neutron diffraction study of the metallic ferromagnet Co3Sn2S2. Solid State Sci..

[20] Murakami S, Kuga S (2008). Universal phase diagrams for the quantum spin Hall systems. Phys. Rev. B.

[21] Okugawa R, Murakami S (2014). Dispersion of Fermi arcs in Weyl semimetals and their evolutions to Dirac cones. Phys. Rev. B.

[22] Liu S, Ohtsuki T, Shindou R (2016). Effect of disorder in a three-dimensional layered Chern insulator. Phys. Rev. Lett..

[23] Hsieh TH, Ishizuka H, Balents L, Hughes TL (2016). Bulk topological proximity effect. Phys. Rev. Lett..

[24] Tuegel, T. I., Chua, V. & Hughes, T. L. Embedded topological insulators. Preprint at arXiv 1802.06790 (2018).

[25] Liu E (2018). Giant anomalous Hall effect in a ferromagnetic kagome-lattice semimetal. Nat. Phys..

[26] Wang Q (2018). Large intrinsic anomalous Hall effect in half-metallic ferromagnet Co3Sn2S2 with magnetic Weyl fermions. Nat. Commun..

[27] Liu DF (2019). Magnetic Weyl semimetal phase in a Kagomé crystal. Science.

[28] Morali N (2019). Fermi-arc diversity on surface terminations of the magnetic Weyl semimetal Co3Sn2S2. Science.

[29] Jiao L (2019). Signatures for half-metallicity and nontrivial surface states in the kagome lattice Weyl semimetal Co3Sn2S2. Phys. Rev. B.

[30] Yin J-X (2019). Negative flat band magnetism in a spin–orbit-coupled correlated kagome magnet. Nat. Phys..

[31] Muechler, L., Liu, E., Gayles, J., Xu, Q., Felser, C. & Sun, Y. Emerging chiral edge states from the confinement of a magnetic Weyl semimetal in Co_3_Sn_2_S_2_. *Phys. Rev. B* **101**, 115106 (2020).

[32] Schnelle W (2013). Ferromagnetic ordering and half-metallic state of Sn2Co3S2 with the shandite-type structure. Phys. Rev. B Condens. Matter Mater. Phys..

[33] Okada Y (2013). Imaging the evolution of metallic states in a correlated iridate. Nat. Mater..

[34] Xing Y (2020). Localized spin-orbit polaron in magnetic Weyl semimetal Co3Sn2S2. Nat. Commun..

[35] Nilius N, Wallis TM, Ho W (2002). Development of one-dimensional band structure in artificial gold chains. Science.

[36] Fölsch S, Hyldgaard P, Koch R, Ploog KH (2004). Quantum confinement in monatomic Cu Chains on Cu(111). Phys. Rev. Lett..

[37] Mugarza A (2002). Lateral quantum wells at vicinal Au(111) studied with angle-resolved photoemission. Phys. Rev. B.

[38] Sagisaka K, Fujita D (2006). Quasi-one-dimensional quantum well on Si(100) surface crafted by using scanning tunneling microscopy tip. Appl. Phys. Lett..

[39] Meyer C, Klijn J, Morgenstern M, Wiesendanger R (2003). Direct measurement of the local density of states of a disordered one-dimensional conductor. Phys. Rev. Lett..

[40] Drozdov IK (2014). One-dimensional topological edge states of bismuth bilayers. Nat. Phys..

[41] Schindler F (2018). Higher-order topology in bismuth. Nat. Phys..

[42] Fujiwara K (2019). Ferromagnetic Co3Sn2S2 thin films fabricated by co-sputtering. Jpn. J. Appl. Phys..

