# Supplementary material for: Evidence for one-dimensional chiral edge states in a magnetic Weyl semimetal Co3Sn2S2
Source: Nat Commun. 2021 Jul 13;12:4269. doi: 10.1038/s41467-021-24561-3 (PMC8277809; doi:10.1038/s41467-021-24561-3)
Supplement: Supplementary file 1 — Supplementary Information [file 41467_2021_24561_MOESM1_ESM.pdf]

Supplemental Information

Evidence for one dimensional chiral edge states in a magnetic Weyl semimetal $Co_3Sn_2S_2$

Sean Howard[1], Lin Jiao[1], Zhenyu Wang[1], Noam Morali[2], Rajib Batabyal[2], Pranab Kumar-Nag[2], Nurit Avraham[2], Haim Beidenkopf[2*], Praveen Vir[3], Enke Liu[3], Chandra Shekhar[3], Claudia Felser[3], Taylor Hughes[4], Vidya Madhavan[1*]

[1]Department of Physics and Frederick Seitz Materials Research Laboratory, University of Illinois Urbana-Champaign, Urbana, Illinois 61801, USA
[2]Condensed Matter Physics Department, Weizmann Institute of Science, 7610001 Rehovot, Israel
[3]Max-Planck-Institute for Chemical Physics of Solids, 01187 Dresden, Germany
[4]Department of Physics and Institute for Condensed Matter Theory, University of Illinois at Urbana-Champaign, Urbana, Illinois 61801, USA

Supplementary Note 1| Theoretical modes for simulating bilayer Chern insulator and 3D WSM.

Bilayer system: We will begin by considering a model having two Chern insulator layers. The Bloch Hamiltonian we will consider is

$$H(\mathbf{k}) = \sin k_x \, I \otimes \sigma^x + \sin k_y \, I \otimes \sigma^y + (m + \cos k_x + \cos k_y) I \otimes \sigma^z + b\tau^x \otimes \sigma^z$$

where τ^a are Pauli matrices in the layer space, and σ^a represent spin. This Hamiltonian has four energy bands with energies

$$\pm E_\pm(\mathbf{k}) = \pm\sqrt{\sin^2 k_x + \sin^2 k_y + (M(\mathbf{k}) \pm b)^2}$$

where $M(\mathbf{k}) = m + \cos k_x + \cos k_y$. The parameter *b* represents the coupling between two layers, and we see that it effectively shifts the mass parameter *m*. For *b* = 0, the system has critical points at *m* = -2, 0, 2 where the Chern number changes its value. For finite *b* there are critical lines in the (*b*, *m*) plane given by $m = -2 \pm b,\ m = \pm b,\ m = 2 \pm b$ as represented in Supplementary Fig. 1a.

Interestingly, when examining the phase diagram, we find regions where the coupling can drive a pair of non-trivial Chern insulators into a completely trivial phase (green shaded region in Supplementary Fig. 1a). This region of the phase diagram has the remarkable feature that a complete bilayer will not exhibit any topological edge states, but a step-like geometry can exhibit a pair of counter-propagating chiral modes. Thus, a trivial stepped bilayer can exhibit a plateau with edge states in a wide swath of parameter space. The idea is that by themselves each layer is topological, but when coupled they are trivial. Consequently, when a part of a layer is removed, the exposed layer below is now “topological” again. The region in Supplementary Fig. 1a that is shaded green is exactly the region where this occurs because the total Chern number is zero and both layers are trivial, but *m* is in the regime where, if *b* was off, the system would be topological. The only thing remaining is to see if this survives when coupled to many layers that act to form a Weyl semimetal.

3D Weyl semimetal: We can make a modification to our bilayer Bloch Hamiltonian to represent a 3D Weyl semimetal. Essentially, the tunneling term between layers needs to be extended to include many layers stacked in the *z*-direction, and we obtain

$$H(\mathbf{k}) = \sin k_x \, \sigma^x + \sin k_y \, \sigma^y + (m + \cos k_x + \cos k_y + \mathrm{b} \cos \mathrm{k_z})\sigma^z$$

where again σ^a represents spin and *b* is the coupling amplitude in the *z*-direction. If *b* is weak, and *m* is tuned so that the layers are nominally in a non-trivial Chern insulator phase, then the system will form a 3D weak topological insulator with sheets of chiral surface states on the *xz* and *yz* surface planes. If *b* is strong enough to close the 2D bulk gap, then the system will form a Weyl semimetal phase with two Weyl nodes separated in the k_z direction. The regions of the phase diagram in the (b, m) plane that represent Weyl semimetal phases are those for which a non-trivial solution of $m + b\cos k_z = -2, 0, 2$ can be found. We thus have the conditions

$$-1 < \frac{-2 - m}{b} < 1$$

$$\text{or } -1 < \frac{-m}{b} < 1$$

$$\text{or } -1 < \frac{2-m}{b} < 1.$$

When at least one of these three conditions are met, the system will have Weyl nodes. If the first (second) [third] condition is met, there will be Weyl nodes at $(k_x, k_y, k_z) = (0, 0, \pm k_z^c), ((\pi, 0, \pm k_z^c), (0, \pi, \pm k_z^c)), [(\pi, \pi, \pm k_z^c)]$ for some value of k_z^c. These are illustrated by the shaded regions in the (b, m) phase diagram in Supplementary Fig. 1b.

Importantly, we see that the region in which we expect a Chern insulator bilayer to exhibit localized modes on step geometries is completely within a Weyl semimetal phase. If we explicitly solve a Weyl semimetal Hamiltonian (*m* = 0.75, *b* = 5) with this step geometry using numerical exact diagonalization, we find a spectrum shown in Supplementary Fig. 1d (calculated for 8 layers) and localized chiral modes on the step plateau as shown in Fig 4a (calculated for 72 layers). Note that these results are qualitatively insensitive to the number of layers used in the numerical model. The modes are localized at the steps but penetrate the layered bulk since the interior is nominally a gapless Weyl semimetal.

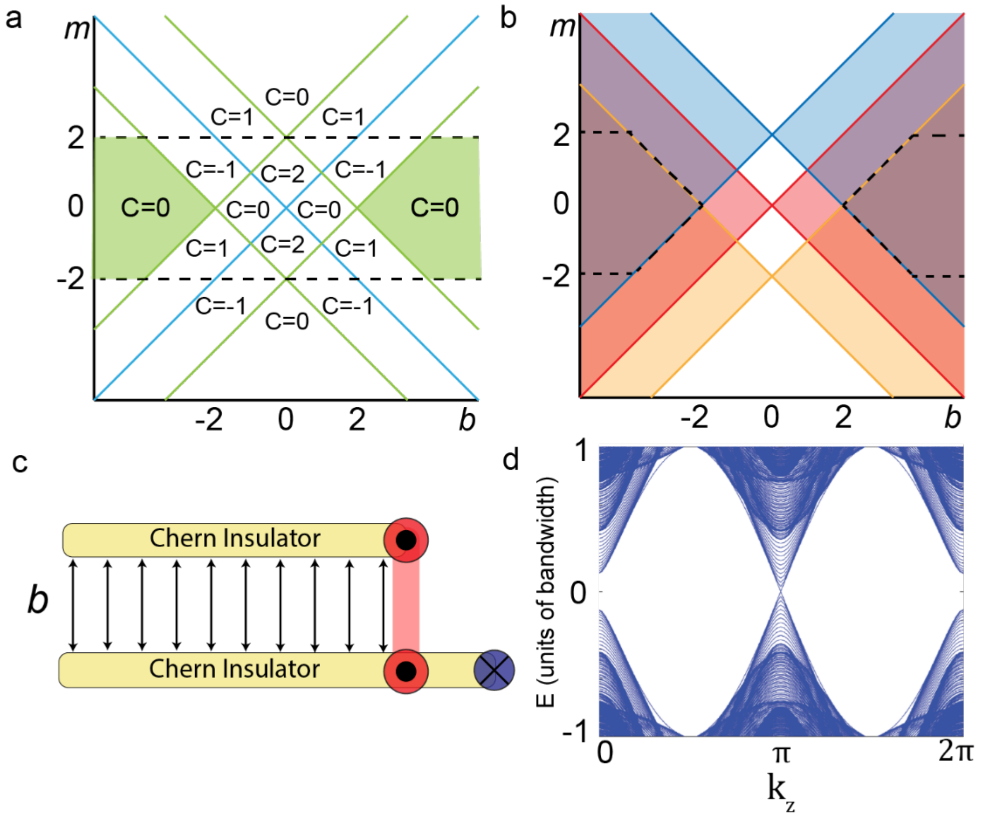

Supplementary Fig. 1 | Model of Chern Insulator Bilayer and Weyl Semimetal. a, Phase diagram for the Chern bilayer system. In the following *b* represents the coupling between two layers and *m* is the mass parameter. The horizontal dashed lines represent the region in which a single layer is a Chern insulator when $b = 0$ and *m* is varied. Diagonal lines represent phase boundaries ($m = \pm b$ is blue, $m = -2 \pm b$ and $m = 2 \pm b$ are green) where the Chern number changes. The green shaded regions indicate coupling where each layer in the bilayer is trivial, but a single layer would be topological without

coupling. **b,** Weyl semimetal phase diagram for a stacking of Chern insulators with coupling. Each of the shaded regions (blue, red, and yellow) represent a condition for Weyl nodes ($-1 < \frac{-2-m}{b} < 1,\ -1 < \frac{-m}{b} < 1, -1 < \frac{2-m}{b} < 1$) being satisfied. **c,** Schematic of the Chern insulator bilayer with a step-like geometry as viewed from the side, showing localized chiral edge modes at the steps. The mode present at the beginning of the bilayer exists on both surfaces due to the coupling. Supplementary Fig. 1d, Dispersion of bands within a 8-layer coupled Chern insulator system (*m* = 0.75, *b* = 5) similar to 72 layer system shown in Fig 4a. The low energy chiral edge modes localized at steps can be seen at *E* = 0, $k_z = \pi$.

Supplementary Note 2 | Determination of Surface Composition from Layer Below Defect Symmetry

For the $Co_3Sn_2S_2$ samples, since cleavage most often occurs between the Sn layer and S layers, the exposed surface imaged with STM will either be the hexagonal S layer with the kagome Co_3Sn layer directly beneath, or hexagonal Sn with hexagonal S directly beneath. As the kagome layer has a different structure compared to the hexagonal S layer, the symmetry of the density of states signatures from defects in the layer below (DLB) can be used to distinguish the Sn surface from the S surface.

DLBs can be identified in the topographies (Supplementary Fig. 2a and Supp. Fig 2e) as extended defects centered in between the top layer atoms. On surfaces with vacancies, we observe large triangular DLB features occupying three lattice sites on each side, showing one vertex that is clearly brighter than the other two (Supplementary Fig. 2b). As seen in the topography, the position of the bright vertex can be different for different defects (labelled 1, 2, 3 in Supplementary Fig. 2a). The distinct bright vertex can also be seen in the real space density of state maps, obtained in the vicinity of these defects (Supplementary Fig. 2d). Considering the layers beneath the S or Sn planes, we realize that only vacancies or substitutional impurities at the Co sites of the Co_3Sn kagome plane have the right symmetry to give rise to the observed density of states signature. This explains both why the whole triangular DLB appears in one angular orientation (i.e., not rotated 60 degrees), and why there are three different bright vertices. On the surface with the adatoms, we do not observe these triangular DLBs. Instead, a "clover" DLB is seen, composed of three equally bright adjacent atoms as shown in Supplementary Fig. 2e,f which persists in DOS maps from -250 mV to 250 mV (Supplementary Fig. 2g,h). The symmetry of these clover DLBs is consistent with a Sn surface showing defects in the S sites in the layer below. With these two pieces of evidence, we unambiguously identify the surface containing vacancies (Fig. 1g) to be the S surface and the surface containing adatoms (Fig. 1h) to be the Sn surface. This determination is of specific importance for future studies of this material, as the nature of Fermi arc states is dependent on the local surface potential.

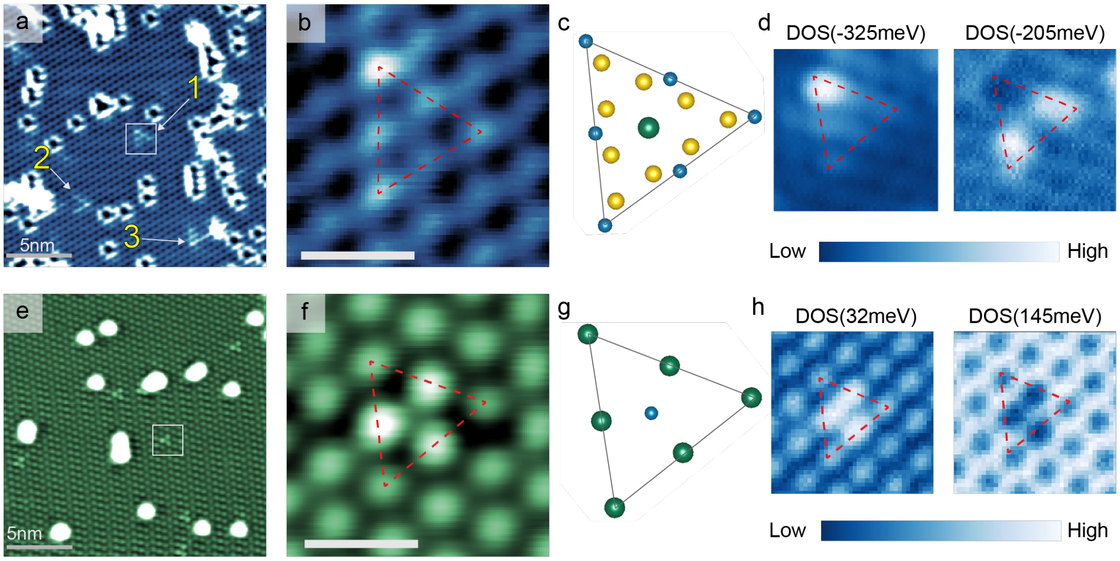

Supplementary Fig. 2 | Topographies and corresponding crystal lattice structure of Sn and S-surfaces.

a, 20 nm x 20nm topography of the surface identified from Fig 1g showing directional triangular DLBs. Three distinct defects with the bright vertex pointing up, right, and left are indicated by numbers 1, 2, and 3 respectively (I = 50 pA, V = -340 mV). **b,** Region within white box in **a** showing a zoomed in triangular DLB. The red dashed line is provided for comparison with **c**. The inset scale bar is 1nm (I = 50 pA, V = -340 mV). **c**, Schematic of the triangle in **b** as the top layer S (blue spheres) and the Co_3Sn layer below. **d,** DOS maps of region in **b** at -325 meV and -205 meV showing that the density of state signature of the triangular DLB has a reduced symmetry compared to two shifted hexagonal layers, indicative of the Co_3Sn layer below (I = 370 pA, V = -350 mV). **e,** 20 nm x 20 nm topography of the surface identified from Fig 1h showing clover DLBs. All DLBs found on this surface were identical. **f,** Region within white box in **e** showing a zoomed in clover DLB. The red dashed line is provided for comparison with **g**. The inset scale bar is 1nm. (I = 50 pA, V = -340 mV). **g,** Schematic of the triangle in **g** as the top layer Sn (green spheres) and the S layer below. **h**, DOS maps of region in **f** at 32 meV and 145 meV showing a trifold symmetry that is consistent with a Sn surface and a S layer below (I = 410 pA, V = -400 mV).

Supplementary Fig. 3 | Point Spectra on S and Sn Step Edges

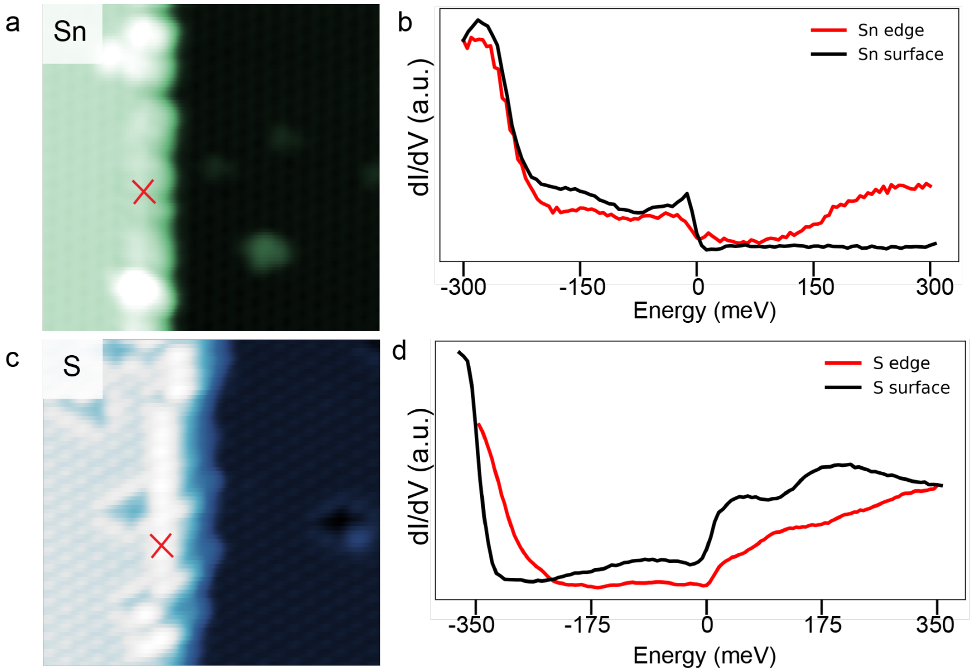

a, Topography (10nm x 10nm) of a Sn step edge. **b,** Comparison of the spectra on the Sn surface to a spectrum on the Sn edge (I = 300 pA, V = -300 mV). The edge spectrum was obtained at the red cross shown in **a**. **c**, Topography (10nm x 10nm) of a S step edge. **d,** Comparison of the spectra on the S surface to a spectrum on the S edge. The edge spectrum was obtained at the red cross shown in **c** (I = 400 pA, V = -400 mV).

Supplementary Fig. 4 | Additional Analysis of Co_3Sn Edge State

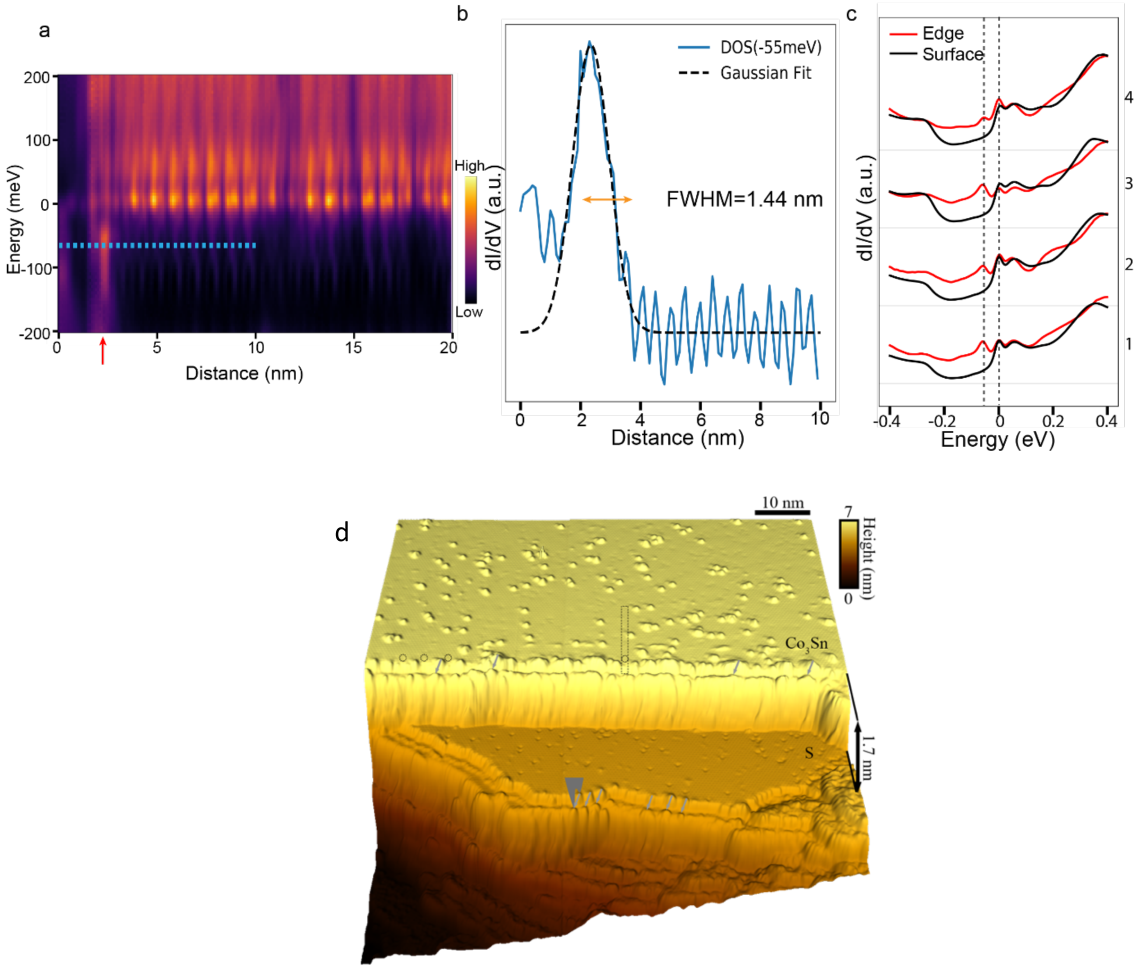

a, Spectroscopic heatmap from Fig. 2c in the main text, with a blue dashed line to show the spatial evolution of the DOS at -55meV in **b**. **b,** Spatial dependence of the DOS at -55 meV across Co_3Sn edge. The peak in the DOS at -55 meV is from the edge state. The peak is fit with a gaussian line shape, and a full width half maximum (FWHM) of 1.44nm is found. This gives us an estimate for the spatial extent of the edge state. **c,** Comparison of four Co_3Sn edge spectra and nearby surface spectra. The spectra labelled 1 are the pair shown in Figure 2 of the main text. All edge spectra show an enhancement of the density of the states below the Fermi energy, peaking at approximately -60 meV, when compared to spectra taken away from the edge. Vertical dashed lines at 0 meV and -60 meV are shown for comparing spectra. **d,** A larger scale image of the Co_3Sn terrace and step where the image in Fig.2a in main text was obtained (dotted area). There are many defects on the edge where these spectra are taken, but no confinement effects are seen in these spectra. This is in contrast to the terrace quantum well like states seen in Figure 3 and Supplemental Information 5, where the close proximity of another edge state allows for hybridization and confinement.

Supplementary Fig. 5 | Two Other Co_3Sn Terraces with Linearly Dispersing Quantum Well Like Bound States

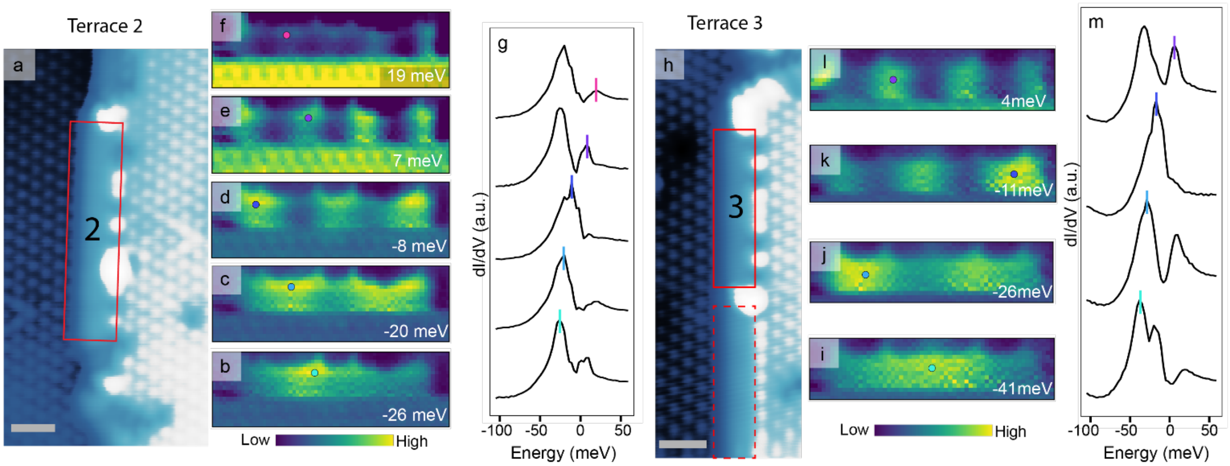

a, Topography of second terrace region showing quantum well like behavior. Scale bar in bottom left corner is 2 nm. Terrace 2 is approximately 6.1 nm long. Two triangular DLBs can be seen both on the top and bottom surface. In general, the terrace length is determined by the spatial extent over which we see the `quantum well like' states (I = 25 pA, V = -200mV). **b-f,** DOS images of n = 1 to n = 5 quantum well like states for terrace 2. Region shown is depicted by red rectangle containing 2 in **a**. Location of spectra shown in **g** are indicated by small colored circles. The color indicates the nth bound state with same scheme as in Fig 3. Note that for n = 1 we used the distance to the left edge to calculate the wavelength due to the presence of an impurity on the right (I = 400 pA, V = -400mV). **g,** Spectra at local density of states maxima indicated in **b-f**. Spectra are offset for clarity. States are represented as circles in Fig 3h. **h**, Topography of two terrace regions showing quantum well like behavior. The red dashed box is same terrace shown in Fig 3a, while the red box containing 3 is the 5 nm long region containing quantum well like states in **i-l.** Scale bar in bottom left corner is 2nm (I = 20 pA, V = -200mV). **i-l,** DOS images from n = 1 to n = 4 in terrace 3. Region shown is depicted by red rectangle containing 3 in **h**. Location of spectra in **m** indicated by small colored circles (I = 200 pA, V = -200mV). **m,** Spectra at local density of states maxima indicated in **i-l**. Spectra are offset for clarity. States are represented by triangles in Fig 3h.

Supplementary Fig. 6 | Spectra and Maps on Single Atom wide Terrace and Comparison to Other Surfaces

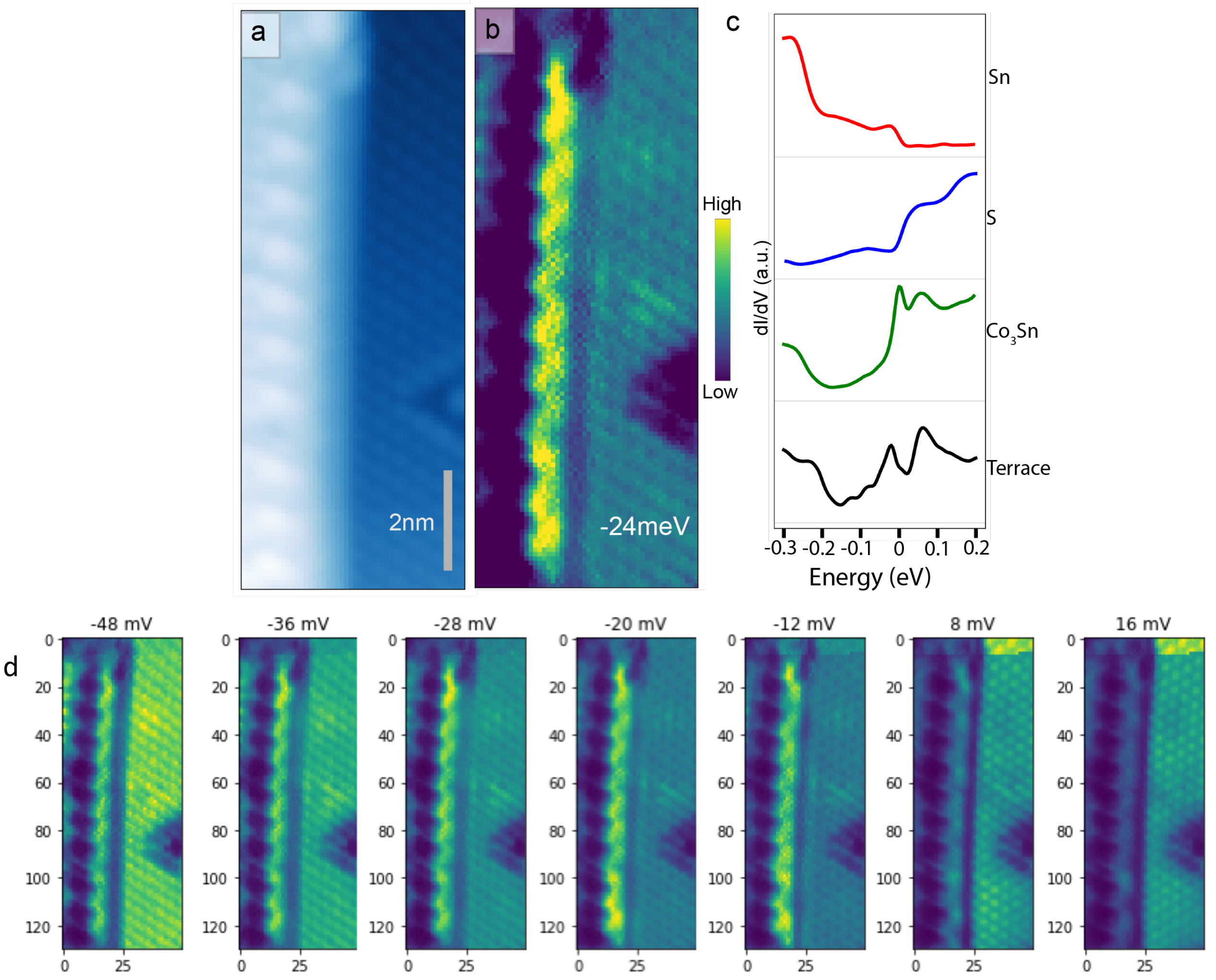

a, 5nm x 13nm topography of a Co_3Sn terrace at a S step edge with only one row of atoms in Co_3Sn layer exposed. Gray 2nm scale bare in the lower right (I = 200 pA, V = -400 mV). **b,** DOS image at -24 mV. The contrast at this bias value allows for better identification of the exposed Co_3Sn layer. **c,** Typical spectra seen on the Sn surface (red), S surface (blue), Co_3Sn surface (green), and the terrace/bright region shown in **b** (black). The spectra are offset for clarity, with a gray line representing zero differential conductance for each spectrum (I = 300 pA, V = -300 mV). **d,** DOS image at a series of voltages showing the distinct lack of quantum well like states (I = 300 pA, V = -300 mV).

Here we report a single row of exposed atoms below a S surface. The single row of atoms is visible as the bright region in the density of states map shown in **b.** While a single row of atoms is not enough to topographically identify this plane, the spectra observed on this terrace (as shown in **c**) most resembles that found on the Co_3Sn plane (also see Fig.2). Unlike terraces observed with larger widths, no quantum well like bound states were observed on this terrace. This is consistent with description of two counter-propagating edge states, since they would annihilate each other if directly on top of one another.

Supplementary Fig. 7 | Layer Determination Based on Terrace Height

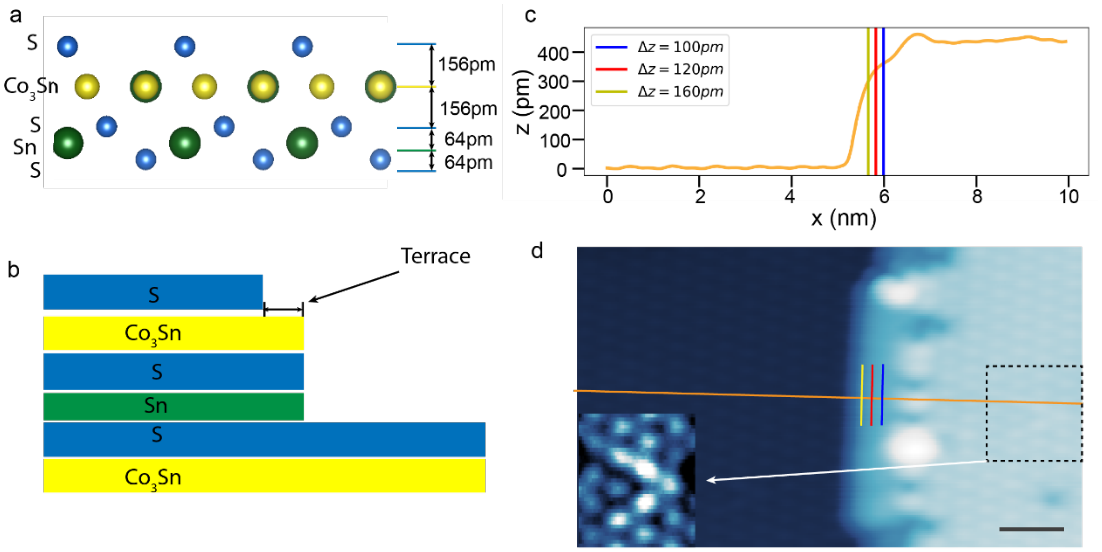

a, Height differences between S, Co_3Sn, and Sn layers. **b,** Schematic of the determined terrace surface composition, with a small thin terrace of Co_3Sn exposed. **c,** Height determination of the terrace from a *z* line profile. Due to the narrow width of the terrace, the measured height changes across the terrace. This is indicated by three colored lines (blue, red, and yellow), which give height changes of 100 pm, 120 pm, and 160pm respectively. **d**, Larger area topography of the terrace where the quantum well like bound states in Terrace 2 were observed. The top layer can be identified from the defects and the spectra as the S plane. The scale bar is 2 nm. (I = 15pA, V = -140 mV). The orange line is the location of the line profile shown in **c**. The blue, red, and yellow lines are the locations along the line profile used in height determination in **c**. The dashed box on the right is the location of a triangular layer below defect shown in the inset. The existence of the triangular layer below defect confirms that the top layer is the S layer with Co_3Sn directly beneath.

Here we perform a detailed analysis of the height change from the S surface to the terrace and find that the height difference observed is approximately 130 pm ± 30 pm. The distances between the S and Co_3Sn layer directly below is 156 pm. Crucially the distance to the next layer, a S layer, is 312 pm which too large to account for the change in height observed. The experimental error in our height determination comes from the thin terrace width as well as density of states contributions.

Supplementary Fig. 8 | Discrete Model of Linearly Dispersing Modes in Finite Quantum Well

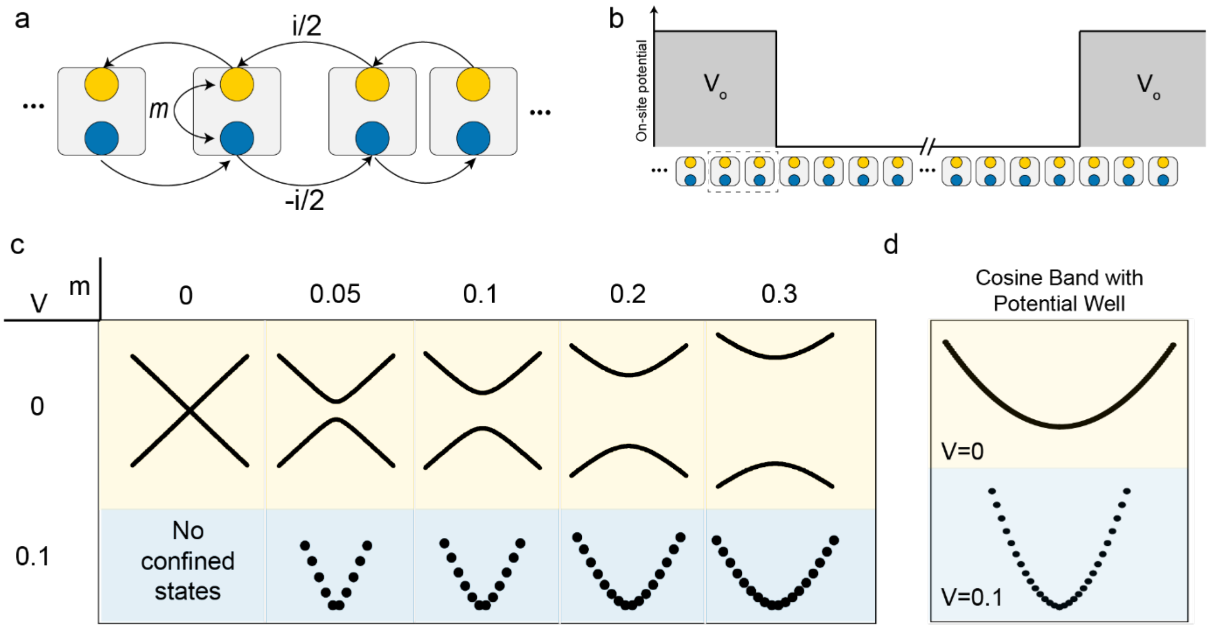

a, Schematic of two unit cells within tight binding Hamiltonian modeling this system: $H = \sum_n \frac{i}{2} c^\dagger_{n+1,\alpha} c_{n,\beta} \sigma^z_{\alpha\beta} - \frac{i}{2} c^\dagger_{n,\alpha} c_{n+1,\beta} \sigma^z_{\alpha\beta} + m c^\dagger_{n,\alpha} c_{n,\beta} \sigma^x_{\alpha\beta} + V(n) c^\dagger_{n,\alpha} c_{n,\beta} \delta_{\alpha\beta}$. This is similar to Fig. 4c, but is reproduced here for context with **b.** A rectangle indicates one unit cell containing two cites represented by yellow and blue circles. Hopping can occur between the same site on adjacent unit cells. The phase relation is such that hopping on one site has positive linear dispersion around zero momentum (yellow circles) while the other site has a negative linear dispersion around zero momentum (blue circles). The sites in one unit cell are allowed to interact via a mixing term m. **b**, Schematic of the potential well $V(n)$**.** An on-site potential much smaller than the bandwidth is applied to states outside the well. For **c** and **d**, a well size of 100 out of 1000 sites is used. **c,** Low momentum band dispersion for the model in **a** for various mixing strengths (light yellow panels) and the states confined within a potential well of 0.1 (light blue panels). For no mixing, no linearly dispersing states are confined within the potential well. As the mixing increases, the low energy and bands become more quadratic and the confined states mimic this behavior. The energy of all states would increase with a potential, as seen in Fig. 4e, although the states outside the well are not shown here. The blue panels are scaled and shifted relative to the yellow panels for easier comparison. **d**, Low momentum band dispersion for a single cosine like band (light yellow panel) and the states confined within a potential well of 0.1 (light blue panel) for comparison with our model.
